# Supplementary material for: Bayesian refinement of protein structures and ensembles against SAXS data using molecular dynamics
Source: PLoS Comput Biol. 2017 Oct 18;13(10):e1005800. doi: 10.1371/journal.pcbi.1005800 (PMC5662244; doi:10.1371/journal.pcbi.1005800)
Supplement: S1 Archive — (BZ2) [file pcbi.1005800.s011.tar.bz2 › gromacs_4.62_bayesian_saxs_refi/share/html/online/g_gyrate.html]

g\_gyrate


| g\_gyrate Main Table of Contents |

**VERSION 4.6  
Sat 19 Jan 2013**

---

### Description

g\_gyrate computes the radius of gyration of a group of atoms
and the radii of gyration about the x-, y- and z-axes,
as a function of time. The atoms are explicitly mass weighted.

With the -nmol option the radius of gyration will be calculated
for multiple molecules by splitting the analysis group in equally
sized parts.

With the option -nz 2D radii of gyration in the x-y plane
of slices along the z-axis are calculated.

### Files

| option | filename | type | description |
| --- | --- | --- | --- |
| **-f** | traj.xtc | Input | Trajectory: xtc trr trj gro g96 pdb cpt |
| **-s** | topol.tpr | Input | Structure+mass(db): tpr tpb tpa gro g96 pdb |
| **-n** | index.ndx | Input, Opt. | Index file |
| **-o** | gyrate.xvg | Output | xvgr/xmgr file |
| **-acf** | moi-acf.xvg | Output, Opt. | xvgr/xmgr file |

### Other options

| option | type | default | description |
| --- | --- | --- | --- |
| **-[no]h** | bool | no | Print help info and quit || **-[no]version** | bool | no | Print version info and quit || **-nice** | int | 19 | Set the nicelevel || **-b** | time | 0 | First frame (ps) to read from trajectory || **-e** | time | 0 | Last frame (ps) to read from trajectory || **-dt** | time | 0 | Only use frame when t MOD dt = first time (ps) || **-[no]w** | bool | no | View output .xvg, .xpm, .eps and .pdb files || **-xvg** | enum | xmgrace | xvg plot formatting: xmgrace, xmgr or none || **-nmol** | int | 1 | The number of molecules to analyze || **-[no]q** | bool | no | Use absolute value of the charge of an atom as weighting factor instead of mass || **-[no]p** | bool | no | Calculate the radii of gyration about the principal axes. || **-[no]moi** | bool | no | Calculate the moments of inertia (defined by the principal axes). || **-nz** | int | 0 | Calculate the 2D radii of gyration of this number of slices along the z-axis || **-acflen** | int | -1 | Length of the ACF, default is half the number of frames || **-[no]normalize** | bool | yes | Normalize ACF || **-P** | enum | 0 | Order of Legendre polynomial for ACF (0 indicates none): 0, 1, 2 or 3 || **-fitfn** | enum | none | Fit function: none, exp, aexp, exp\_exp, vac, exp5, exp7, exp9 or erffit || **-ncskip** | int | 0 | Skip this many points in the output file of correlation functions || **-beginfit** | real | 0 | Time where to begin the exponential fit of the correlation function || **-endfit** | real | -1 | Time where to end the exponential fit of the correlation function, -1 is until the end |

---

http://www.gromacs.org  
gromacs@gromacs.org
